# Nutrition, metabolism, and epigenetics: pathways of circadian reprogramming

**DOI:** 10.15252/embr.202152412

**Published:** 2022-04-12

**Authors:** Tomoki Sato, Paolo Sassone‐Corsi

**Affiliations:** ^1^ Department of Biological Chemistry Center for Epigenetics and Metabolism School of Medicine INSERM U1233 University of California Irvine CA USA; ^2^ Present address: Laboratory of Nutritional Biochemistry Graduate School of Nutritional and Environmental Sciences University of Shizuoka Shizuoka Japan

**Keywords:** circadian clock, energy metabolism, epigenetics, nutrition, Chromatin, Transcription & Genomics, Metabolism

## Abstract

Food intake profoundly affects systemic physiology. A large body of evidence has indicated a link between food intake and circadian rhythms, and ~24‐h cycles are deemed essential for adapting internal homeostasis to the external environment. Circadian rhythms are controlled by the biological clock, a molecular system remarkably conserved throughout evolution. The circadian clock controls the cyclic expression of numerous genes, a regulatory program common to all mammalian cells, which may lead to various metabolic and physiological disturbances if hindered. Although the circadian clock regulates multiple metabolic pathways, metabolic states also provide feedback on the molecular clock. Therefore, a remarkable feature is reprogramming by nutritional challenges, such as a high‐fat diet, fasting, ketogenic diet, and caloric restriction. In addition, various factors such as energy balance, histone modifications, and nuclear receptor activity are involved in the remodeling of the clock. Herein, we review the interaction of dietary components with the circadian system and illustrate the relationships linking the molecular clock to metabolism and critical roles in the remodeling process.

## Graphical abstract

The intake of food and its composition affect the circadian clock. This review provides a comprehensive overview of the interconnections between the nutritional status, metabolic homeostasis and circadian rhythms.

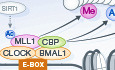

**Glossary**
ACC: acetyl‐CoA carboxylase
AceCS1: acetyl‐CoA synthetase 1
ACLY: ATP‐citrate lyase
AMP: adenosine monophosphate
AMPK: 5' AMP‐activated protein kinase
Angptl8: angiopoietin‐like 8
ATP: adenosine triphosphate
BMAL1: brain and muscle ARNT‐like 1
cAMP: cyclic adenosine monophosphate
CCGs: clock‐controlled genes
CLOCK: circadian locomotor output cycles protein caput
CREB: cAMP response element‐binding protein
CRY: cryptochrome
DBP: D site of albumin promoter binding protein
DEC1: differentiated embryo chondrocyte 1
E4bp4: E4 promotor‐binding protein 4
E‐box: enhancer box
EGCG: epigallocatechin gallate
GCs: glucocorticoids
GPC: sn‐glycero‐3‐phosphocholine
GR: glucocorticoid receptor
GSK3β: glycogen synthase kinase 3β
HAT: histone acetyl transferase
HDAC: histone deacetylases
HFD: high‐fat diet
HMGCS2: 3‐hydroxy‐3‐methylglutaryl‐CoA synthase 2
JMJD2B: jumonji C domain‐containing protein 2B
KD: ketogenic diet
LKB1: serine/threonine kinase liver kinase B1
LXR: liver X receptor
MAPK: mitogen‐activated protein kinase
MLL1: mixed lineage leukemia 1
mTOR: mammalian target of rapamycin
NAD⁺: oxidized nicotinamide adenine dinucleotide
NADH: reduced nicotinamide adenine dinucleotide
NAM: nicotinamide mononucleotide
NAMPT: nicotinamide phosphoribosyltransferase
Npy: neuropeptide Y
O‐GlcNAc: O‐linked N‐acetyl‐glucosamine
O‐GlcNAcylation: O‐linked N‐acetylglucosamine modification
OGT: O‐linked GlcNAc transferase
PC: phosphatidylcholine
Per: period
PGC‐1α: peroxisome proliferator‐activated receptor‐gamma coactivator‐1α
Pomc: pro‐opiomelanocortin
PPAR: peroxisome proliferator‐activated receptor
RBP4: retinol‐binding protein 4
ROR: RAR‐related orphan receptor
SAM: s‐adenosyl methionine
SCN: suprachiasmatic nucleus
SIRT: sirtuin
SREBP‐1c: sterol‐regulatory element‐binding protein‐1c
STAT5: signal transducer and activator of transcription 5
TRF: time‐restricted feeding
UDP‐GlcNAc: uridine diphosphate N‐acetylglucosamine
β‐OHB: β‐hydroxybutyrate

## Introduction

Every morning, after a night of sleep, we wake up, eat our regularly timed meals, go through our normal routines, sleep, and then repeat the same cycle. Various physiological functions, including sleep and being awake, body temperature, hormone secretion, locomotor activity, and appetite, are regulated by an autonomous, ~24‐h mechanism, termed the circadian clock (Sahar & Sassone‐Corsi, 2012). This endogenous timekeeper allows organisms to anticipate daily environmental fluctuations and time internal processes (Bass & Takahashi, 2010; Eckel‐Mahan & Sassone‐Corsi, 2013). Anatomically, the mammalian central clock or pacemaker is located in the suprachiasmatic nucleus (SCN) of the hypothalamus, with functions regulated by photic inputs from the retina in the form of light. Notably, light‐induced resetting of the SCN clock depends on wavelength. Blue light (380–500 nm) potentially exerts more robust effects on mammalian circadian rhythms than green and yellow wavelengths (Lockley *et al*, 2003). A transformative discovery around the turn of the century revealed that in addition to the brain, the circadian clock functions in peripheral organs, including the liver and muscle (Schibler & Sassone‐Corsi, 2002). These local or peripheral clocks are semi‐autonomous elements of a larger system and are synchronized by the SCN clock, functioning as an “orchestra director,” via neural, hormonal (e.g., glucocorticoids [GCs], insulin, and melatonin), and behavioral inputs (Saini *et al*, 2011).

**Figure 1:**
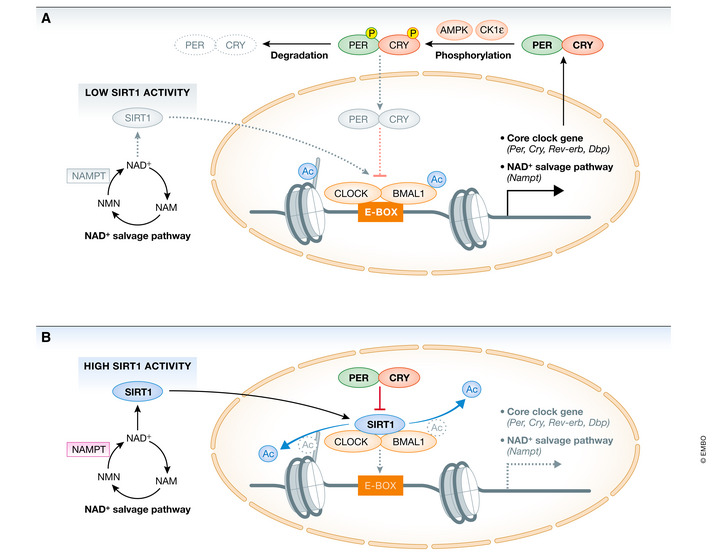
The molecular organization of the circadian clock (A) Daytime: The core transcription factor BMAL1 heterodimerizes with CLOCK to form a CLOCK:BMAL1 complex. CLOCK acetylates BMAL1 and histone tails, leading to chromatin opening that promotes binding of CLOCK:BMAL1 to E‐box elements in promoter regions of the core‐clock genes and clock‐controlled genes (*Per, Cry, Rev‐erv*, *Nampt*). During the daytime AMPK and CK1ε contribute to phosphorylation and degradation of the negative regulators CRY and PER, respectively, thus relieving the negative feedback on CLOCK:BMAL1. The circadian activity of SIRT1, which regulates cyclic acetylation levels of BMAL1 and histones in nucleosomes associated with clock‐controlled genes, is controlled by the rhythmic cellular levels of its cofactor NAD⁺. *Nampt* gene expression and cellular NAD⁺ levels oscillate and peak at night, leading to lower SIRT1 activity during the daytime. (B) Nighttime: PER and CRY protein accumulate in the cytosol during the night, heterodimerize and translocate to the nucleus to repress CLOCK:BMAL1 transcriptional activity. SIRT1 deacetylase activity during the nighttime is high, deacetylating BMAL1 and histone tails. SIRT1‐mediated histone deacetylation induces packing of DNA (heterochromatin) and gene silencing. AMPK, 5′ AMP‐activated protein kinase; BMAL1, brain and muscle ARNT‐like 1; CK1ε, casein kinase 1 epsilon; CLOCK, circadian locomotor output cycles protein caput; CRY, cryptochrome; NAD⁺, oxidized nicotinamide adenine dinucleotide; *Nampt*, nicotinamide phosphoribosyltransferase; PER, period; SIRT1, sirtuin 1.

The molecular machinery underlying the circadian clock consists of a transcriptional/translational feedback loop: the core transcription factors, brain and muscle Arnt‐like 1 (BMAL1) and circadian locomotor output cycles kaput (CLOCK), heterodimerize and drive the expression of core clock genes or output genes by binding to enhancer boxes (E‐boxes) on target promoters (Fig 1) (Crane & Young, 2014). As E‐boxes are among the most common promoter elements in the genome, the clock can transcriptionally control a large array of genes. In addition, CLOCK:BMAL1 directly activates the transcription of *Period* (*Per1*, *Per2*, and *Per3*) and *Cryptochrome* (*Cry1* and *Cry2*) genes, known to encode transcriptional repressors that dimerize and generate a tightly regulated negative portion of the feedback loop (Gekakis *et al*, 1998; Kume *et al*, 1999; Shearman *et al*, 2000; Lee *et al*, 2001; Padmanabhan *et al*, 2012; Kim *et al*, 2014). An additional level of circadian regulation involves the nuclear receptors, i.e., RAR‐related orphan receptors (RORs) and REV‐ERBα (Nr1d1), which activate and repress *Bmal1* transcription, respectively (Reppert & Weaver, 2002; Everett & Lazar, 2014; Partch *et al*, 2014). Furthermore, virtually all clock proteins are reportedly regulated by post‐translational modifications, including phosphorylation, acetylation, ubiquitination, and O‐linked N‐acetylglucosamine modification (O‐GlcNAcylation) (Asher & Schibler, 2011; Kaasik *et al*, 2013; Li *et al*, 2013).

Numerous studies have highlighted how the clock system closely interacts with energy metabolism in peripheral organs (Bass, 2012; Eckel‐Mahan *et al*, 2013). Although the SCN clock contributes to circadian variations in glucose homeostasis via glucose uptake and insulin release, peripheral clocks can also constitute another layer of regulation of these processes. For example, melatonin secreted from the pineal gland in an SCN clock‐dependent manner directly regulates pancreatic insulin secretion (Peschke *et al*, 1997; Picinato *et al*, 2002). Pancreatic insulin secretion is regulated by multisynaptic projections from the SCN (Ueyama *et al*, 1999; Buijs *et al*, 2001). In addition to insulin‐mediated signaling, it has been suggested that the SCN may regulate glucose uptake in peripheral organs through the nervous system (La Fleur, 2003). Moreover, the clock machinery controls the expression of numerous metabolic output genes in peripheral tissues (Dibner *et al*, 2010; Maury *et al*, 2010). Accordingly, genetic disruption of mouse clock components can induce metabolic diseases, including obesity, by attenuating rhythmic changes in hormone concentration and metabolic gene expression (Dibner *et al*, 2010; Maury *et al*, 2010). In humans, disruption of circadian rhythms owing to jet lag, time shift work, and irregular meal timing has been linked to metabolic diseases (Kettner *et al*, 2015; Morris *et al*, 2016). These observations suggest that the circadian clock controls several signaling pathways encompassing major components of metabolic homeostasis.

Accumulated evidence has suggested that the quality and timing of meals markedly alter circadian metabolism (Eckel‐Mahan *et al*, 2013; Tognini *et al*, 2017). For example, a comparison of oscillating transcripts in different tissues revealed that approximately 15% of hepatic transcripts and 4% of muscle transcripts oscillate under *ad libitum* conditions of a normal chow diet (Hughes *et al*, 2010; Zhang *et al*, 2014b). A high‐fat diet (HFD), wherein energy from fat exceeds 40%, can disrupt normally oscillating genes and induce *de novo* oscillations (Eckel‐Mahan *et al*, 2013). Transcriptome analyses have revealed that a large fraction of the genome can be potentially controlled by the clock, and diet‐induced remodeling exerts tissue‐specific effects (Masri & Sassone‐Corsi, 2010). Moreover, varying the macronutrient composition and specific nutrients (nobiletin, resveratrol, and caffeine) have been reported to influence peripheral clock gene expression (Sherman *et al*, 2011; Sun *et al*, 2015; He *et al*, 2016).

In this review, we describe the relationship between the circadian clock and metabolic homeostasis from the perspective of energy balance and epigenome regulation. We focus on the effect of dietary composition on the molecular clock and discuss how nutritional approaches may contribute to the prevention of metabolic diseases.

## Reciprocal regulation between the circadian clock and metabolites

According to several genome‐wide expression studies, genes involved in glucose metabolism, lipid metabolism, heme biosynthesis, and mitochondrial adenosine triphosphate (ATP) synthesis all exhibit a circadian pattern of expression (Ceriani *et al*, 2002; Panda *et al*, 2002; Oishi *et al*, 2003; Gachon *et al*, 2004). Importantly, several transcriptional factors central to regulating metabolic pathways have diurnal gene expression patterns and activity. Peroxisome proliferator‐activated receptors (PPAR) compose a group of nuclear receptor proteins that play essential roles in regulating glucose and lipid metabolism (Oishi *et al*, 2005; Canaple *et al*, 2006). Transcription of *Pparα*, a PPAR isotype, can be activated by CLOCK:BMAL1 via an intronic E‐box‐rich region. In contrast, it has been reported that *Pparγ* expression may be regulated by the products of two clock‐controlled genes, the D site albumin promoter binding protein (Dbp) and E4 promoter‐binding protein 4 (E4bp4). DBP and E4BP4 jointly induce the circadian expression of one *Pparγ* subtype by binding to D‐box sequences located in the first exon of the gene (Takahashi *et al*, 2010), thus indicating that Pparγ may be regulated by the circadian clock component. RORα plays a crucial role in the homeostasis of lipid metabolism in the liver by negatively regulating PPARγ signaling via histone deacetylase (HDAC) 3 recruitment to *Pparγ* target promoters (Kim *et al*, 2017). However, it remains unclear whether RORα regulates other PPAR isotypes.

REV‐ERBα directly regulates the transcription of genes controlling carbohydrate and lipid metabolism by binding to ROR‐responsive elements via its DNA‐binding domain (Zhang *et al*, 2015). Furthermore, REV‐ERBα exhibits a DNA‐binding domain‐independent function that modulates liver metabolism (Zhang *et al*, 2015). Based on experiments in cultured hepatocytes, downregulation of *Rev‐erbα* by siRNA increases the expression of *glucose‐6‐phosphatase (G6Pase)* and *phosphoenolpyruvate carboxykinase (Pepck)*, which are rate‐limiting enzymes of gluconeogenesis (Yin *et al*, 2007). In addition, the administration of REV‐ERB agonists alters the rhythm and amplitude of metabolic genes in the liver, skeletal muscles, and adipose tissue (Solt *et al*, 2012).

Apart from direct transcriptional control, diverse studies have revealed that the clock control of metabolism is pervasive and multilayered. High‐throughput metabolomic investigations of human plasma and saliva samples have revealed that approximately 15% of all identified metabolites oscillate in a circadian manner (Minami *et al*, 2009; Dallmann *et al*, 2012). Notably, a high proportion of rhythmic metabolites in blood plasma are lipid metabolites, such as fatty acids and phospholipids. As outputs of the circadian system, some metabolites also provide feedback and operate as inputs to the circadian clock. Oxidized nicotinamide adenine dinucleotide (NAD⁺) is one such example. The circadian clock controls NAD⁺ levels by transcriptionally regulating the *nicotinamide phosphoribosyltransferase (Nampt)* gene, which encodes an enzyme that catalyzes the rate‐limiting step in the NAD⁺ salvage pathway (Fig 1B). The activator complex CLOCK:BMAL1 directly binds to the *Nampt* promoter to control its circadian expression, leading to the oscillation of NAD⁺ levels from recycled nicotinamide mononucleotide (NAM). Importantly, NAD⁺ operates as a coenzyme for Class III histone deacetylases and sirtuins (SIRTs), and its cyclic accumulation results in rhythmic deacetylation of SIRT targets, ultimately contributing to the circadian gene expression (Nakahata *et al*, 2008; Feng *et al*, 2011). We provide the detailed interaction mechanism between SIRT1 and the circadian clock system in the section on “Energy balance controls the clock and metabolic homeostasis”.

Several lipid metabolites, including phospholipids and free fatty acids, also contribute to circadian rhythmicity. PPARs are activated by dietary fatty acids and their metabolic derivatives, thus serving as lipid sensors that activate lipid metabolism, including lipogenesis and lipolysis. Liu *et al* (2013) reported that plasma levels of phosphatidylcholine (PC) 36:1 fluctuate diurnally and that PC production is regulated by hepatic PPARδ (Liu *et al*, 2013). Tandem mass spectrometry scanning identified PC 36:1 as PC (18:0/18:1), which acts as a ligand for PPARα in muscle, producing a rhythm of fatty acid uptake (Liu *et al*, 2013); this finding suggests that rhythmic oscillations of lipid metabolites derived from hepatic lipogenesis can integrate metabolic functions between the liver and muscle. PPARs are associated with sterol element‐binding protein‐1c (SREBP‐1c), a master regulator that controls hepatic *de novo* lipogenesis. Recent studies have shown that a sn‐glycero‐3‐phosphocholine (GPC) (16:0/18:1), presumed to be a ligand of PPARα, is produced in an SREBP‐1c‐dependent manner, establishing a circadian rhythm for lipid oxidation in the liver (Guan *et al*, 2018).

Collectively, these findings illustrate that NAD⁺ and lipid pathways are both outputs and inputs to the clock mechanism. The concept that specific metabolites influence clock function implies that consuming diets containing these nutrients, or their precursors, may enable genomic and metabolic reprogramming.

## Energy balance controls the clock and metabolic homeostasis

The circadian clock system is essential for adapting to and anticipating the surrounding environment. Notably, it contains certain factors that act as sensors to recognize the nutritional state. The intracellular ratios of adenosine monophosphate and triphosphate (AMP/ATP) and NAD⁺/reduced nicotinamide adenine dinucleotide (NADH) reflect the nutritional and energetic states. Several studies have revealed that 5′ AMP‐activated protein kinase (AMPK) and SIRT1 act as nutritional sensors to relay metabolic information to the clock. For example, AMPK is activated under low‐energy conditions, as reflected by a high AMP/ATP ratio. Activated AMPK enhances catabolic processes to restore intracellular ATP levels. AMPK phosphorylation at Thr172 is required for AMPK activation, and serine/threonine kinase liver kinase B1 (LKB1) directly mediates this event (Hawley *et al*, 2003; Liang *et al*, 2007). AMPK activity was reportedly rhythmic in the mouse liver, hypothalamus, and fibroblasts (Um *et al*, 2011). Notably, AMPK activation can influence the circadian clock system by reducing the stability of the core clock repressors CRY1 and PER2, and thus, may contribute to the metabolic entrainment of clocks in peripheral tissues (Fig 1A). AMPK directly phosphorylates the serine (S)71 and S280 residues of CRY1 and enhances its degradation; mutation of either S71 or S280 to a non‐phosphorylatable amino acid (alanine) blocks this effect (Lamia *et al*, 2009). Phosphorylated CRY1 exhibits lower affinity for PER2 and instead increases binding to *F‐box and Leu‐rich repeat protein 3* (*FBXL3*), a ubiquitin ligase that promotes CRY1 ubiquitination and degradation (Gatfield & Schibler, 2007). Furthermore, AMPK phosphorylates casein kinase 1ε (CK1ε), resulting in periodic phosphorylation and degradation of PER2 (Um *et al*, 2007). AMPK deletion leads to tissue‐specific alterations in circadian gene expression (Um *et al*, 2011). Basic helix‐loop‐helix transcription factor differentiated embryo chondrocyte 1 (*Dec1*) is a clock gene that acts on the negative limb by suppressing CLOCK and BMAL1 transcriptional activity. DEC1 protein expression can be inversely correlated with AMPK activity and negatively regulates AMPK activity via LKB1 (Sato *et al*, 2015). According to one report, the kinetics of metformin‐induced AMPK activation in the liver appeared remarkably dependent on circadian time (Henriksson *et al*, 2017), indicating that AMPK is clock‐controlled. AMPK regulates clock components, as well as directly phosphorylates cooperating transcription factors that play key roles in lipid metabolism. Activated AMPK phosphorylates SREBP‐1c, leading to SREBP‐1c protein degradation and suppression of *de novo* lipogenesis in the liver (Lee *et al*, 2015). Acetyl‐CoA carboxylase (ACC) is another AMPK target; ACC phosphorylation decreases malonyl‐CoA levels, thereby activating carnitine palmitoyltransferase 1 (CPT 1) and resulting in fatty acid oxidation (Fullerton *et al*, 2013).

SIRT1 is a sensor of energy balance that bridges circadian regulation and nutritional status. SIRT1 activity is modulated by the cellular redox state, which can be inferred from the NAD⁺/NADH ratio (Asher *et al*, 2008). SIRT1 regulates several metabolic processes, including gluconeogenesis, insulin sensitivity, and *de novo* lipogenesis via the deacetylation of various proteins and histones. Several SIRT1‐regulated transcription factors are known to be involved in nutrient flux, such as peroxisome proliferator‐activated receptor‐gamma coactivator‐1α (PGC‐1α), AMPK, and forkhead box O1 (FOXO1) (Schwer & Verdin, 2008). The deacetylase activity of SIRT1 is synchronized with the diurnal variation of NAD⁺, inducing rhythmic activity in target proteins. Recent studies have suggested that SIRT1 regulates the acetylation level of acetyl‐CoA synthetase 1 (AceCS1), which is localized in the cytoplasm and is required for cytosolic acetyl‐CoA metabolism, and may affect intracellular acetyl‐CoA levels and the total amount of acetylated protein (Sato *et al*, 2017). In addition, SIRT1 deacetylates and regulates several clock proteins and acts as a key modulator of the circadian clock machinery (Asher *et al*, 2008; Nakahata *et al*, 2008). SIRT1 interacts with the CLOCK:BMAL1 complex in a circadian fashion, inducing diurnal fluctuations in transcriptional activity (Fig 1B). Additionally, SIRT1‐mediated deacetylation of PER2 is critical for the stability of PER2 (Asher *et al*, 2008). A mutant SIRT1 was shown to enhance BMAL1 acetylation, concomitant with higher amplitude of circadian gene expression (Foteinou *et al*, 2018), and loss of SIRT1 reportedly led to a lower amplitude of the circadian rhythms in SIRT1 knockout mouse embryonic fibroblast cells (Asher *et al*, 2008). Recent computational and experimental approaches have supported PER2, not BMAL1, as the direct target of SIRT1 (Foteinou *et al*, 2018). Moreover, the authors suggested that SIRT1‐induced the deacetylation of PGC‐1α enhances ROR‐driven BMAL1 transcription via PGC‐1α coactivation.

The SIRT1 and AMPK signaling pathways are tightly coordinated (Ruderman *et al*, 2010). For example, AMPK increases the cellular NAD^+^/NADH ratio, which subsequently activates the deacetylase activity of SIRT1 (Canto *et al*, 2009). Conversely, SIRT1 controls AMPK activation via deacetylation of the AMPK‐activating kinase LKB1 (Hou *et al*, 2008; Lan *et al*, 2008). Therefore, AMPK and SIRT1 not only regulate each other, but their metabolic actions often converge, indicating that their close interrelationship might also play a role in circadian clock regulation (Ruderman *et al*, 2010).

AMPK and SIRT1 are sensors that detect energy deficiency, whereas the mammalian mechanistic target of rapamycin (mTOR) is a nutrient sensor that detects over‐nutrition. Notably, the mTOR signaling pathway is activated by growth factors (e.g., insulin and insulin‐like growth factor‐1) and amino acids (leucine and arginine) (Inoki *et al*, 2002; Nair & Short, 2005). In response to these extracellular signals, mTOR regulates various physiological processes, including cell growth, protein synthesis, and autophagy (Morita *et al*, 2013). mTOR protein expression and activity exhibit robust circadian rhythms (Cao *et al*, 2011). The expression of F‐box and WD repeat domain‐containing 7 (Fbxw7), a ubiquitin ligase that degrades mTOR protein, is transcriptionally regulated by DBP (Cao *et al*, 2011). The circadian clock system regulates mTOR, and conversely, mTOR directly affects the period and amplitude of the circadian clock at the cellular level. Ramanathan *et al* (2018) revealed that mTOR activation accelerates clock oscillations, whereas inhibition lengthens the circadian period in both the SCN and peripheral tissues (Ramanathan *et al*, 2018). Regulating BMAL1 can partly clarify the effect of mTOR on the CLOCK system. Reportedly, insulin‐mediated activation of mTOR‐ribosomal protein S6 kinase signaling can inhibit the nuclear localization of BMAL1 by phosphorylating S42 (Dang *et al*, 2016). Moreover, Cao *et al* reported that disruption of mTOR signaling attenuates the light‐induced phase delay of circadian locomotor activity (Cao *et al*, 2010). These findings suggest that mTOR may also function as a hub for peripheral and central clock regulation by external stimuli, such as insulin and light.

For intracellular proteins, O‐GlcNAcylation, a post‐translational modification incorporating O‐linked N‐acetyl‐glucosamine (O‐GlcNAc) into specific serine/threonine residues of proteins, is a key mediator of the metabolic response to nutrient availability (Hart, 2019). This widespread and dynamic glycosylation is mediated by O‐linked GlcNAc transferase (OGT) and O‐GlcNAcase (OGA), catalyzing sugar addition and removal, respectively (Yang & Qian, 2017; Hart, 2019). O‐GlcNAcylation depends on the concentration of uridine diphosphate N‐acetylglucosamine (UDP‐GlcNAc), the donor for O‐GlcNAcylation, produced via the hexosamine biosynthesis pathway and glucose flux. As the production of UDP‐GlcNAc requires glucose as a precursor, O‐GlcNAcylation is recognized as a cellular glucose sensor. OGT can modify numerous proteins, including key transcription factors of glucose and lipid metabolism, such as liver X receptor (LXR) and carbohydrate‐responsive element‐binding protein (ChREBP) (Anthonisen *et al*, 2010; Bindesboll *et al*, 2015). In addition, several core clock genes are known targets of O‐GlcNAcylation. O‐GlcNAcylation competitively inhibits the phosphorylation of PER and CRY, increasing their stabilization (Kim *et al*, 2012; Kaasik *et al*, 2013). O‐GlcNAcylation also stabilizes CLOCK:BMAL1, promoting target gene expression (Li *et al*, 2013). A recent study has revealed that REV‐ERBα, but not REV‐ERBβ, interacts with cytoplasmic and nuclear OGT. Moreover, the diurnal fluctuation of REV‐ERBα protein has been linked to rhythms of OGT protein stability and protein O‐GlcNAcylation (Berthier *et al*, 2018).

## Nutritional control of epigenome modifications

**Figure 2:**
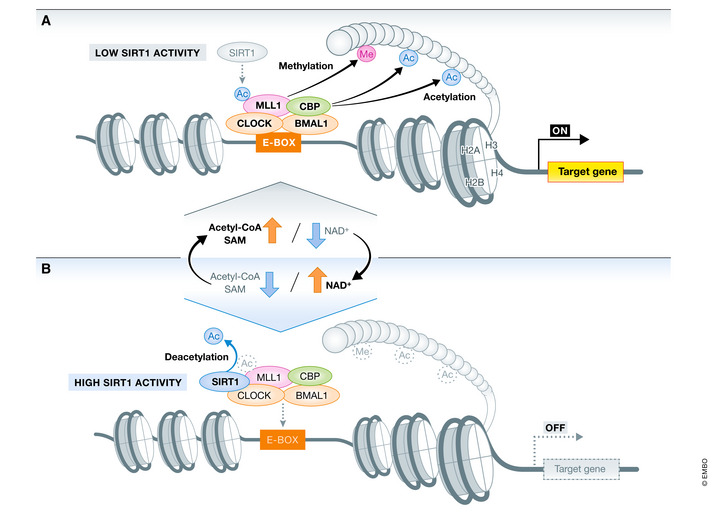
Nutritional conditions affect the chromatin landscape Most chromatin‐modifying enzymes use intermediary metabolites as cofactors or substrates, and their activity is regulated by the availability of these metabolites. Acetyl‐CoA, SAM, and NAD⁺ are substrates for histone acetylation, methylation, and deacetylation, respectively. Acetyl‐CoA is necessary for protein acetylation. SAM is an important methyl‐group donor metabolite in DNA and histone methylation. SIRT1 deacetylates histone lysine residues using NAD^+^ as a co‐substrate. (A) MLL1 physically interacts with CLOCK and contributes to CLOCK:BMAL1 recruitment to the E‐box regions. Together, MLL1 and CLOCK induce histone modifications (H3K4me4, H3K9Ac, and H3K14Ac) and enhance target gene expression. (B) Oscillating levels of NAD⁺ control SIRT1 deacetylase activity (histone and non‐histone proteins). Under high cellular NAD⁺ conditions, SIRT1 deacetylates MLL1, an event that reduces MLL1 enzymatic activity and leads to decreased H3K4me3 and H3K9/14ac levels. NAD⁺ thus controls MLL1 acetylation level and CLOCK:BMAL1 dependent transcription. BMAL1, brain and muscle ARNT‐like 1; CLOCK, circadian locomotor output cycles protein caput; MLL1, mixed‐lineage leukemia 1; NAD⁺, oxidized nicotinamide adenine dinucleotide; SAM, s‐adenosyl methionine; SIRT1, sirtuin 1.

Molecular signatures of epigenetic regulation and chromatin architecture are fundamental to genetically determined biological processes. Chromatin histone modifications, such as acetylation, methylation, and phosphorylation, regulate the chromatin structure and control gene expression (Fig 2). For example, histone H3 Lys27 (H3K27ac) acetylation indicates active gene transcription, whereas trimethylation of the same residue (H3K27me3) leads to gene silencing. Over the past decades, accumulating evidence has undoubtedly established that cellular metabolism exerts a profound and dynamic influence on histone modifications, as histone‐modifying enzymes utilize key metabolic intermediates.

As a precursor of anabolic reactions and acetyl group donor for acetylation of histone and non‐histone proteins, acetyl‐CoA is critical in cellular processes. The nuclear/cytosolic pool of acetyl‐CoA is maintained by two enzymes, AceCS1 and ATP‐citrate lyase (ACLY) (Albaugh *et al*, 2011). Acetate and citrate are metabolized to acetyl‐CoA by AceCS1 and ACLY, respectively. A decrease in acetyl‐CoA levels due to loss of both enzymes reportedly reduces global histone acetylation (Wellen *et al*, 2009; Ariyannur *et al*, 2010). Histone modification is reversible, and histone acetylation is removed by histone deacetylases (HDACs), which are components of transcriptional repressor complexes. SIRT1 catalyzes a deacetylation reaction that requires NAD⁺. NAD⁺ can be synthesized *de novo* from diverse dietary sources (e.g., tryptophan) or regenerated from NAM via the salvage pathway (Canto *et al*, 2015; Verdin, 2015). Transcript and protein levels of SIRT1 in peripheral tissues remain constant during day and night, but SIRT1 enzymatic activity is regulated by the availability of NAD⁺ (Fig 2) (Nakahata *et al*, 2008, 2009).

S‐adenosyl methionine (SAM) is a universal substrate for DNA methylation and methylation of arginine and lysine residues of histone and non‐histone proteins. SAM is synthesized from methionine and ATP by methionine adenosyltransferase (Grillo & Colombatto, 2008). After methyl group transfer by SAM‐dependent methylase, S‐adenosyl homocysteine, a byproduct of the reaction, acts as a potent methyltransferase inhibitor (Selhub & Miller, 1992). The importance of dietary methyl donors in epigenetic regulation has been examined in rodent models and humans, and the effects of maternal dietary intake have been the focus of recent studies (Donohoe & Bultman, 2012; Huang *et al*, 2014; Shorter *et al*, 2014).

The molecular clock hinges on epigenetic mechanisms. CLOCK itself has an intrinsic histone acetyltransferase (HAT) activity necessary for circadian function (Doi *et al*, 2006). The HAT activity of CLOCK is preferentially linked to targets H3K9 and H3K14. In addition, CLOCK acetylates its binding partner, BMAL1, a crucial event for circadian rhythmicity. Other proteins with HAT activity, such as CBP and P300, can associate with CLOCK:BMAL1 and participate in rhythmic histone acetylation (Curtis *et al*, 2004). SIRT1 is known to competitively regulate acetylation marks on CLOCK targets, such as H3K9 and H3K14, as well as K537 of BMAL1 (Nakahata *et al*, 2008).

According to Katada and Aguilar‐Arnala, a key event in circadian transcriptional activation is the interaction of CLOCK:BMAL1 with proteins associated with the Set1/COMPASS complex component mixed‐lineage leukemia 1 (MLL1), whose enzymatic activity results in the transcription‐activating histone mark H3K4me3 (Katada & Sassone‐Corsi, 2010; Aguilar‐Arnal *et al*, 2015). MLL1 and CLOCK physically interact at specific circadian times, paralleling the cyclic peaks of transcription. The H3K4me3 modification catalyzed by MLL1 favors the recruitment of CLOCK:BMAL1 to chromatin and subsequent H3K9/K14 acetylation (Fig 2).

## The feeding‐fasting cycle remodels the clock and metabolic homeostasis

The circadian clock and metabolic homeostasis are regulated by the cellular environment, including energy and hormone balance. The energy balance and diurnal rhythmicity of blood hormone concentrations depend on feeding behavior and dietary composition. It has been established that feeding‐fasting cycles are potent timing cues for the clock in peripheral tissues. Mice primarily consume food during the night (active phase), consuming 20% of their daily caloric intake during the day (Bare, 1959; Koronowski *et al*, 2019). To experimentally clarify the feeding‐fasting cycle in mice, it is necessary to restrict food consumption by time‐restricted feeding (TRF). Disturbance of the feeding‐fasting cycle by restricted daytime feeding gradually inverts the phase of peripheral clocks (Damiola, 2000). Intriguingly, the impact of restricted daytime feeding on the SCN clock phase is considered negligible.

Fasting affects clock‐controlled genes (CCGs) and fasting‐sensitive transcription factors, and it suppresses CLOCK:BMAL1 recruitment to the E‐box region of *Dbp* and *Rev‐erbα*, consequently diminishing diurnal gene expression of these genes (Kinouchi *et al*, 2018). Under fasting conditions, the amount of cellular NAD⁺ is increased while acetylated BMAL1 protein is decreased, resulting in diminished transcriptional activity of CLOCK:BMAL1. Glucagon, a hormone secreted by pancreatic α cells under fasting conditions, induces the hepatic expression of *Per1* and *Per2* genes through the cyclic adenosine monophosphate (cAMP) response element‐binding protein (CREB) signaling pathway (Mukherji *et al*, 2015). In addition, Kinouchi *et al* reported that fasting activates transcription factors such as CREB and glucocorticoid receptor (GR) (Kinouchi *et al*, 2018). However, the authors revealed that *Per2* expression was reduced under fasting conditions. This discrepancy is likely attributed to different fasting durations (12 h vs. 24 h) and possibly the timing of fasting initiation across studies. Moreover, fasting increases cellular levels of AMP, enhancing AMPK activation. Activated AMPK phosphorylates and destabilizes CRY1 protein. Collectively, these results suggest that fasting greatly remodels metabolic homeostasis by decreasing CLOCK:BMAL1 transcriptional activity and controlling metabolic gene expression by fasting‐induced transcription factors.

Feeding triggers an insulin surge that prompts the liver to switch to anabolic processes such as glycogenesis and *de novo* lipogenesis. Numerous studies have reported that insulin is a potent hormone regulating peripheral clock function, but not insulin‐independent tissues (Balsalobre *et al*, 2000; Yamajuku *et al*, 2012; Sato *et al*, 2014). Administration of insulin reportedly increased the hepatic expression of *Per2* via the phosphatidylinositol 3‐kinase (PI3K) pathway and induced a phase shift of the clock (Yamajuku *et al*, 2012). In adipose tissues, insulin stimulated *Per2* expression via the mitogen‐activated protein kinase (MAPK) pathway, resulting in a phase shift in the adipose clock similar to that observed in the liver (Sato *et al*, 2014). BMAL1 is also an essential regulator of insulin‐induced phase resetting and promotes lipid metabolism via the action of insulin (Zhang *et al*, 2014a). *Bmal1* deficiency inhibited AKT phosphorylation by reducing the abundance of rapamycin‐insensitive companion of mammalian target of rapamycin (RICTOR), a key component of mTOR complex 2, and attenuated insulin signaling. In *Bmal1*‐depleted mice, refeeding failed to activate insulin signaling and increase gene expression related to the clock and *de novo* lipogenesis. Recently, it has been reported that in addition to insulin, oxyntomodulin, a peptide hormone released from the gastrointestinal tract after feeding, induces the expression of *Per1* and *Per2* in the liver (Landgraf *et al*, 2015). Based on these reports, insulin and oxyntomodulin appear to play important roles in the timing and output of peripheral clocks.

Glycogen synthase kinase 3β (GSK3β) has been proposed to play a critical role in insulin signaling (Wan *et al*, 2013). GSK3β is a ubiquitous kinase that regulates diverse cellular processes, including glucose homeostasis. Notably, GSK3β exhibits a circadian pattern of activity, and BMAL1 phosphorylation by this kinase controls its stability (Sahar *et al*, 2010). Moreover, GSK3β reportedly phosphorylates PER2, CRY2, and REV‐ERBα, leading to proteasomal degradation of CRY2 and stabilization of REV‐ERBα (Harada *et al*, 2005; Yin *et al*, 2006). O‐GlcNAc modification competitively inhibits the phosphorylation of PER and CRY proteins and GSK3β phosphorylates OGT. Thus, the circadian activity of GSK3β contributes to the development of a diurnal pattern of protein O‐GlcNAcylation (Kaasik *et al*, 2013; Li *et al*, 2013). Thus, GSK3β is involved in the tuning of the clock by post‐translational modification of its proteins.

Currently, the effects of hepatokines on the clock and metabolic homeostasis are gaining momentum (Ma *et al*, 2016; Chen *et al*, 2019). The liver is sensitive to food signals and responds via cytokine secretion. Chen *et al* (2019) reported that the secretion of angiopoietin‐like 8 (Angptl8) is a potential direct link between food intake, hepatic clock resetting, and metabolic gene transcription (Chen *et al*, 2019). Angptl8 induces the phosphorylation of P38 MAPK, nuclear factor‐κB (NF‐κB), and AKT, ultimately activating *Per1* expression. In addition, retinol‐binding protein 4 (RBP4), a specific retinol carrier in the circulation (Quadro *et al*, 1999), is produced and released mainly by hepatocytes. RBP4 oscillates in a daily manner under *Dbp* control. Ma *et al* (2016) revealed that RBP4 acts as a hepatokine in the temporal regulation of glucose metabolism (Ma *et al*, 2016).

## Clock remodeling by dietary composition

The rhythm of peripheral clocks can be maintained by normal chow TRF, independent of central clock synchronization (Chaix *et al*, 2018). In certain metabolic tissues, daytime‐restricted feeding results in 12‐h phase shifts of peripheral circadian clock gene expression (Vollmers *et al*, 2009; Bray *et al*, 2013). Thus, while meal timing is an important factor, dietary composition is also important for guiding the clock system. Several studies have demonstrated the molecular mechanisms of clock remodeling and circadian metabolism based on dietary composition. Herein, we review the effects of high fat and ketogenic diets (KD).

### How does a high‐fat diet alter the circadian clock system and energy metabolism?

**Figure 3:**
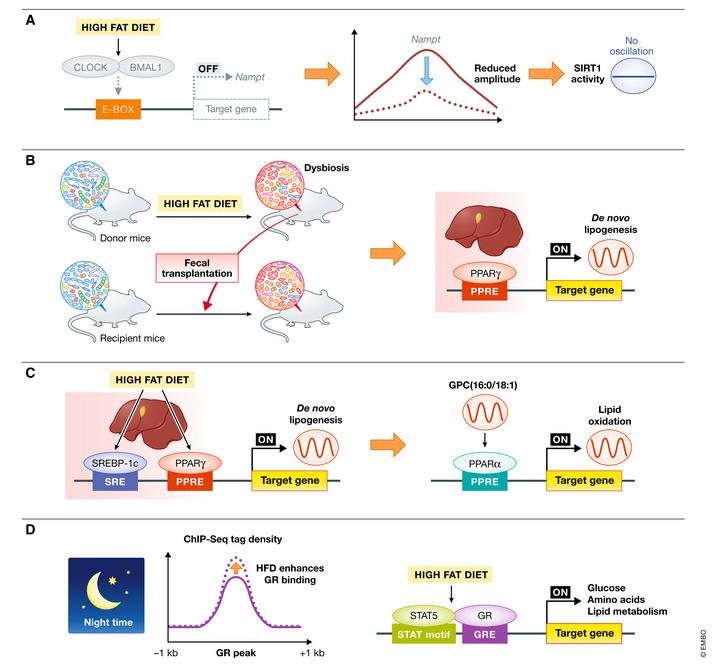
High‐fat diet (HFD)‐induced reprogramming of the hepatic clock and metabolic homeostasis (A) HFD blunts CLOCK:BMAL1 chromatin recruitment to the promoter region of *Nampt,* dampening transcriptional oscillation and as a consequence the oscillation of SIRT1 activity. (B) HFD treatment induces dysbiosis. When chow‐diet fed mice receive fecal transplantation from HFD‐treated mice, the PPARγ pathway is activated in their liver in a ZT‐dependent manner, resulting in transcriptional reprogramming. (C) HFD leads to a remarkable and synchronous circadian oscillation of the anabolic transcriptional factors SREBP‐1c and PPARγ in the liver, leading to rhythmic *de novo* lipogenesis. SREBP‐1c elevates the levels of the PPARα ligand sn‐glycero‐3‐phosphocholine (GPC) (16:0/18:1). Rhythmic GPC (16:0/18:1) level induce circadian hepatic fatty acid oxidation. (D) HFD increases GR binding near promoters and enhances GR‐STAT5 co‐occupancy in target genes related to glucose, amino acid, and lipid metabolism. BMAL1, brain and muscle ARNT‐like 1; CBP, histone acetyltransferase CREB‐binding protein; CLOCK, circadian locomotor output cycles protein caput; Nampt, nicotinamide phosphoribosyltransferase; GR, glucocorticoid receptor; PPARα, peroxisome proliferator‐activated receptor‐alpha; PPARγ, peroxisome proliferator‐activated receptor‐gamma; SREBP‐1c, sterol‐regulatory element‐binding protein‐1c; STAT5, signal transducer and activator of transcription 5.

A typical rodent HFD contains 60% kcal of energy as lipids. Chronic HFD intake induces metabolic diseases, such as obesity, insulin resistance, and diabetes. Recent studies have revealed massive changes in circadian gene expression in diet‐induced obesity (Kohsaka *et al*, 2007; Eckel‐Mahan *et al*, 2013). How does HFD remodel the oscillation of peripheral gene expression? To address this question, Eckel‐Mahan *et al* (2013) analyzed the liver metabolome and transcriptome profile using high‐throughput “omics” analysis (Eckel‐Mahan *et al*, 2013). HFD induced the loss of oscillation in a large number of normally oscillating genes. Core‐clock genes are maintained even in HFD‐fed animals, indicating their high resistance to dietary composition. In contrast, HFD induced large‐scale *de novo* oscillating transcripts. HFD‐induced reprogramming is mediated by several mechanisms (Fig 3). Recruitment of CLOCK:BMAL1 to chromatin is impaired in genes that would normally be considered clock‐controlled. HFD decreased the transcriptional activity of CLOCK and BMAL1 without affecting their mRNA and protein expression (Fig 3A). Another study reported that HFD severely impaired the amplitude and rhythmicity of clock genes in the liver (Wang *et al*, 2018). Both experiments used the same HFD (60% fat, D12491 Research Diets, Inc.), but the administration period differed (10 weeks vs. 12 weeks). Recruitment of CLOCK:BMAL1 to E‐box regions of target genes decreased within three days of HFD administration. Further examination is required to reveal the complete course of acute and chronic effects of HFD on the clock.

In addition, PPARγ is involved in HFD‐mediated metabolic remodeling. HFD enhanced nuclear PPARγ protein levels and rhythmic chromatin recruitment to target genes (Eckel‐Mahan *et al*, 2013). In liver‐specific PPARγ‐deficient mice, the expression of numerous genes involved in lipid uptake and lipid transport decreased remarkably, resulting in reduced hepatic steatosis (Moran‐Salvador *et al*, 2011). RORα specifically recruited HDAC3 to PPARγ target promoters and suppressed PPARγ transcriptional activity (Kim *et al*, 2017). HFD administration to liver‐specific RORα‐deficient mice induced severe steatosis and obesity by dysregulating PPARγ signaling (Kim *et al*, 2017). In addition, HFD regulated PPARγ gene expression through epigenetic modifications. The histone demethylase Jumonji C domain‐containing protein 2 B (JMJD2B) is involved in HFD‐induced PPARγ gene expression (Kim *et al*, 2018). Overexpression of JMJD2B increased the expression of PPARγ and caused hepatic steatosis. In addition, studies have demonstrated that histone H3K4 methyltransferase MLL4 is recruited to the peroxisome proliferator response elements of PPARγ and target genes in the liver, an event linked with steatosis (Kim *et al*, 2016). Moreover, it has been recently reported that HFD regulates the circadian clock in the liver by altering the gut microbiota. To investigate the direct effect of the microbiome on the hepatic clock, Murakami *et al* (2016) colonized control chow‐fed recipient mice with microbial communities harvested from HFD‐fed donors and analyzed the circadian clock machinery in the liver (Murakami *et al*, 2016). The authors demonstrated that fecal transplantation from HFD to chow mice induced rhythmic activation of hepatic PPARγ, which, in turn, leads to transcriptional reprogramming in the liver (Fig 3B). Additionally, in mice whose microbiome was ablated by antibiotic treatment, HFD disrupted the PPARγ rhythm and target gene expression. These results strongly indicate that PPARγ is involved in HFD‐induced steatosis and that hepatic lipid accumulation can be prevented by suppressing the PPARγ network. In contrast, Guan *et al* (2018) reported that SREBP‐1c is the primary factor for HFD‐induced metabolic remodeling (Fig 3C) (Guan *et al*, 2018). The authors reported that *Pparγ* deletion caused no significant changes in the amplitude or rhythmicity of hepatic *de novo* lipogenesis genes. HFD‐induced SREBP‐1c oscillations have been observed in other HFD studies as well. SIRT1 reportedly deacetylates SREBP‐1c and inhibits its activity (Wang *et al*, 2017). As described above, HFD inhibits *Nampt* transcription by attenuating CLOCK:BMAL1 transcriptional activity, which in turn lowers SIRT1 activity. This links HFD‐induced clock remodeling with the regulation of SREBP‐1c‐dependent *de novo* lipogenesis. HFD‐induced SREBP‐1c oscillations also leads to circadian production of GPC (16:0/18:1) and circadian fatty oxidation in the liver (Fig 3C).

In addition to PPARγ and SREBP‐1c, GR has recently been reported as a nuclear receptor responsible for HFD‐induced metabolic adaptations (Quagliarini *et al*, 2019). GR mediates most of the known biological effects of GCs and steroid hormones secreted by the zona fasciculata of the adrenal cortex. GC secretion exhibits a prominent circadian rhythm and peaks during feeding time, i.e., the early night in rodents and early morning in humans (Weitzman *et al*, 1971; Windle *et al*, 1998). GCs are GR ligands, and activated GRs are translocated to the nucleus to regulate target gene expression. The promoter region of *Per* contains a GR‐binding region, and *Per* gene expression rapidly increases after GR activation (Yamamoto *et al*, 2005; So *et al*, 2009). Subsequently, GR represses the transcriptional level of *Rev‐erba* (Torra *et al*, 2000). GR binding to target promoter regions exhibits a distinct daily pattern, closely mirroring serum corticosterone levels (Quagliarini *et al*, 2019). A 12‐week HFD administration maintained the typical circadian GR binding pattern while expanding the GR cistrome, especially during the feeding phase in the liver (Quagliarini *et al*, 2019). Simultaneously, the recruitment of signal transducer and activator of transcription 5 (STAT5), a transcriptional factor that genetically interacts with GR in liver homeostasis, is also increased, and the HFD‐mediated increase in GR occupancy is driven by STAT5 (Fig 3D). This finding suggests that GR is one of the factors inducing HFD‐mediated reprogramming. Other nuclear receptors, hepatocyte nuclear factor‐4α (HNF‐4α) and LXR, have also been reported to play a role in regulating the clock system (Noshiro *et al*, 2009; Qu *et al*, 2018), and concurrently, both nuclear receptors are involved in the homeostasis of carbohydrate and lipid metabolism (Joseph *et al*, 2002; Rhee *et al*, 2003; Gilardi *et al*, 2014). During HFD feeding, metabolic remodeling involves the interaction of numerous nuclear receptors.

HFD blunts feeding‐fasting cycles in mice, increases daily food consumption, and affects the circadian patterns of circulating hormones (Kohsaka *et al*, 2007; Hatori *et al*, 2012; Vieira *et al*, 2012). Leptin plays a major role in regulating appetite via mediobasal hypothalamic signaling (Begg & Woods, 2013). Reportedly, blood leptin levels exhibit circadian rhythmicity and are subjected to acute regulation by food intake. HFD increased blood leptin concentration (Sundaram & Yan, 2016). Hypothalamic neuropeptides linked to feeding are known to determine eating behavior. The saturated fatty acid palmitate, a largely abundant lipid, was shown to increase the expression of *neuropeptide Y (Npy)* and *pro‐opiomelanocortin (Pomc)* (Clemenzi *et al*, 2020). Other *in vitro* experiments have indicated that palmitate treatment enhanced *Bmal1* and reduced *Per2* in neuronal cells (Fick *et al*, 2011; Greco *et al*, 2014). BMAL1 rhythmically binds to *Npy* and *Pomc* promoters and increases *Npy* and *Pomc* gene expression (Fick *et al*, 2010; Loganathan *et al*, 2019). As palmitate treatment‐induced *Npy* and *Pomc* expression diminished in *Bmal1* knockout neuronal cells, a palmitate‐BMAL1‐NPY axis in the hypothalamus may contribute to HFD‐altered feeding patterns (Clemenzi *et al*, 2020).

### How does a ketogenic diet change the circadian clock system and energy metabolism?

**Figure 4:**
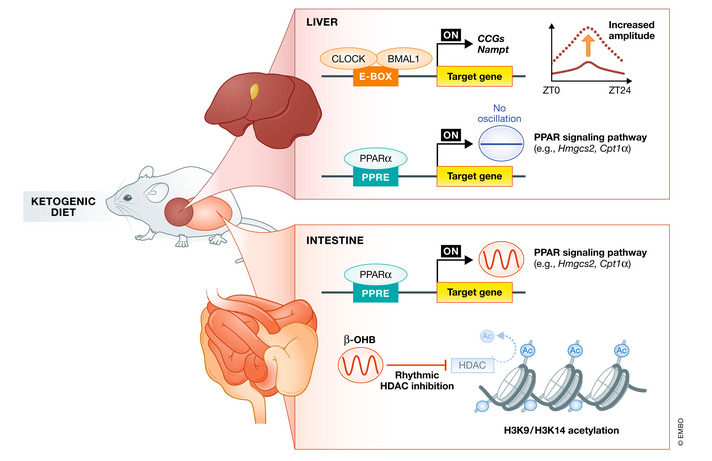
Ketogenic diet (KD)‐induced reprogramming of peripheral clocks and metabolic homeostasis KD remodels rhythms differently in the liver and intestine. KD enhances BMAL1 recruitment to target genes in the liver, thereby increasing the amplitude of CCGs oscillations. KD activates PPARα signaling both in the liver and intestine. However, robust oscillations can be observed only in the intestine. KD‐induced ketogenesis increases serum β‐OHB concentration and circadian oscillations. The local intestinal concentration of β‐OHB mirrored the profile of serum β‐OHB, leading to time‐dependent HDAC activity and histone acetylation. BMAL1, brain and muscle ARNT‐like 1; β‐OHB, β‐hydroxybutyrate; CCGs, clock‐controlled genes; CPT‐1α, carnitine palmitoyltransferase 1α; HDAC, histone deacetylases; HMGCS2, 3‐hydroxy‐3‐methylglutaryl‐CoA synthase 2; PPARα, peroxisome proliferator‐activated receptor alpha.

In contrast to HFD, KD is a high‐fat diet with low carbohydrate and low protein content. KD has primarily been used for weight loss in obese individuals (Bueno *et al*, 2013; Nymo *et al*, 2017). Indeed, KD consumption can induce a switch to fatty acid oxidation and provide excess acetyl‐CoA, resulting in ketone body generation, including acetoacetate and β‐hydroxybutyrate (β‐OHB). KD mediates different effects on the liver and intestine circadian clocks (Fig 4) (Tognini *et al*, 2017). In the liver, administration of KD for 4 weeks enhanced the amplitude of genes with an E‐box sequence in the promoter region, such as *Dbp*, *Nampt*, and *patatin‐like phospholipase domain‐containing 2* (*Pnpla2*) and increased the recruitment of BMAL1 to E‐boxes. Genome‐wide analysis showed that 2,339 genes displayed circadian expression in the liver when compared with 719 genes under normal dietary conditions. In contrast, oscillation of only 785 novel genes was observed in the intestine. Transcriptomic analysis revealed that the PPARα transcriptional network was enriched in KD‐fed mice. Intriguingly, although the PPARα pathway was induced both, in the liver and in the intestine during ketogenesis, the two tissues displayed different oscillation phases for PPARα nuclear accumulation and target gene expression. Serum and gut levels of β‐OHB, an endogenous HDAC I inhibitor (Shimazu *et al*, 2013) were increasingly correlating with increased histone H3 acetylation levels in the gut but not the liver, which might contribute to the oscillation of PPARα target genes in the gut. In addition, KD increased global protein acetylation levels in whole‐cell lysates and the mitochondrial fraction isolated from the liver (Newman *et al*, 2017). Recent evidence indicates a more direct epigenetic effect of β‐OHB via a novel histone modification: β‐hydroxybutyrylation of H3K9 (Xie *et al*, 2016). Further studies are required to clarify the effects of KD on histone modification.

### Differences in metabolic reprogramming between HFD and KD

Both HFD and KD regimens contain a high‐fat component (HFD 60%, KD 90%) but differ in carbohydrate and protein content. KD contains < 1% carbohydrate and 10% protein, mimicking caloric restriction. Following the administration of KD and HFD, a transcriptional analysis identified the downregulation of multiple metabolic pathways such as insulin signaling and protein synthesis, common to KD and HFD (Newman *et al*, 2017). However, the effects on PPARα signaling differed between these two studies. Newman *et al* (2017) reported the upregulation of PPARα target genes unique to KD (Newman *et al*, 2017). However, Guan *et al* (2018) showed that HFD induced diurnal oscillation of PPARα (Guan *et al*, 2018). This discrepancy could be attributed to differences in diet composition and administration methods in each study. HFD and KD have different effects on the energy balance. Low glucose conditions, such as calorie restriction and fasting, enhanced AMPK activation (Zhu *et al*, 2005; Zhang *et al*, 2013). KD mimicked calorie restriction and resulted in the activation of AMPK. In contrast, HFD decreased hepatic (but not muscle) AMPK phosphorylation levels (Shiwa *et al*, 2015), which can be reversed by implementing a normal diet for three days (Shiwa *et al*, 2015). Another major difference between HFD and KD groups was the blood β‐OHB levels. After one week of dietary intervention, the blood β‐OHB concentration tended to be higher in the KD group than in the HFD group, and the value after 6 weeks was significantly higher in the KD group (Roberts *et al*, 2017). Mitochondrial 3‐hydroxy‐3‐methylglutaryl‐CoA synthase 2 (HMGCS2) is a key enzyme in ketogenesis (Puisac *et al*, 2012). It has been reported that insulin repressed the mRNA and protein expression of HMGCS2 (Nadal *et al*, 2002). As the blood insulin level under KD was markedly reduced when compared with that under HFD (Kennedy *et al*, 2007), HMGCS2 expression is expected to be higher, and the increase in β‐OHB concentration could also be attributed to differences in insulin concentration. As mentioned above, β‐OHB can inhibit HDAC activity. After one month of KD, total acetyl‐Lys levels were individually increased by 5‐ and 2.5‐fold in the liver and muscle of the KD‐treated group when compared with the HFD‐treated group (Roberts *et al*, 2017). Differences in comprehensive protein acetylation levels may therefore be one mechanism that induces differences in metabolic characteristics between the two diet groups and the difference in the carbohydrate content is one of the key factors resulting in this difference between HFD and KD.

### How do carbohydrates affect the circadian clock system and energy metabolism?

Glucose is widely accepted as one of the factors that can influence cellular circadian rhythms (Hirota *et al*, 2002). However, most studies that examined the effect of carbohydrates on clock remodeling and metabolic homeostasis have been performed *in vitro* using cultured cells, and knowledge regarding its *in vivo* effects remain limited.

Hirota *et al* (2002) demonstrated that exchanging the culture medium induced circadian gene expression in cultured rat fibroblasts and that glucose is a key molecule that can directly reset the phase of cells derived from peripheral tissues (Hirota *et al*, 2002). Glucose reportedly increases expression of the *transforming growth factor beta‐inducible early gene 1* (*Tieg1*) and transiently accumulates TIEG1 protein in the nucleus, which directly represses *Bmal1*expression, resulting in reduced *Per1* and *Per2* levels (Hirota *et al*, 2010).

Fructose and glucose are characterized by the same chemical formula; however, they differ in structure and metabolism. In hepatocytes, fructose led to disrupted *Bmal1* mRNA expression and delayed the expression of *Per1* mRNA (Chapnik *et al*, 2016). In myotubes, fructose induced a higher amplitude of *Per1* and *Bmal1* mRNA expression and delayed rhythms of *Per1*, *Bmal1*, and *Clock* (Chapnik *et al*, 2016). Based on these *in vitro* results, the influence of fructose on clock genes varies between organs and corresponding tissues.

AMPK activation is considered one of the factors responsible for differences in glucose‐ and fructose‐mediated effects on the molecular mechanism of the circadian clock. The phosphorylated AMPK/AMPK ratio in hepatocytes treated with fructose was approximately half that of glucose‐treated hepatocytes (Chapnik *et al*, 2016). On the other hand, fructose exposure increased the myocyte p‐AMPK/AMPK ratio by approximately 2.5‐fold when compared with that following glucose exposure (Chapnik *et al*, 2016). Thus, the effects of glucose and fructose on AMPK are tissue‐specific. As AMPK is involved in the degradation of CRY (Lamia *et al*, 2009), differences in the glucose‐ and fructose‐induced effects on clock molecules may be mediated by AMPK. In addition to the direct effects of carbohydrates on organs, it is necessary to consider the effects of hormones secreted following carbohydrate intake. Insulin is strongly involved in metabolic changes in peripheral organs after carbohydrate intake.

Glucose is a potent stimulant of insulin secretion; however, fructose intake either marginally or fails to increase the insulin concentration (Sato *et al*, 2019). Thus, to clarify the effects of carbohydrate intake on clock function in organisms, it is necessary to consider the effects of carbohydrates, as well as the associated effects of changes in hormone concentrations.

In contrast to the findings described above, four weeks of a high‐sucrose diet did not alter hepatic clock gene expression in rats (Sun *et al*, 2019a). Conversely, it modestly changed the amplitude and rhythm of gene expression related to fructolysis and *de novo lipogenesis*, leading to fatty liver and the development of hyperlipidemia. Hepatic phosphate levels were reduced under high‐sucrose diet, indicating lower ATP levels (Sun *et al*, 2019a). Since ATP is a putative mRNA stabilizer (Chen *et al*, 2007), these reports imply that variations in ATP‐dependent RNA stability contribute to the high‐sucrose‐induced amplitude enhancement of lipogenic enzyme mRNA in the liver.

In contrast to the liver, a four‐week high‐sucrose diet altered the expression of nutrient transporters and carbohydrate metabolism, as well as the oscillation patterns of circadian clock genes in the small intestine (Sun *et al*, 2019b). These results indicate that sucrose treatment has a greater impact on the intestinal clock and metabolism than the liver.

In mice experiments, each carbohydrate (glucose, fructose, and sucrose) causes distinct shifts in circadian timing in the liver (Hirao *et al*, 2009). Further studies in animal models are required to elucidate the precise mechanisms by which carbohydrates influence peripheral clock function *in vivo*.

## Positive impact of clock remodeling on health and metabolism

It is widely established that circadian misalignment leads to obesity and several metabolic diseases, such as insulin resistance, dyslipidemia, and hyperglycemia (Shimba *et al*, 2011; Paschos *et al*, 2012; Shi *et al*, 2013). Conversely, these findings suggest that maintaining proper circadian rhythms can result in health benefits. Eating behavior is one of the most influential factors in maintaining a proper circadian clock. TRF, a feeding strategy that limits daily food intake, prolongs the temporary fasting time and forms a clear feeding/fasting cycle, enhancing the robustness or amplitude of rhythms. In mice, TRF during the dark phase can improve several detrimental metabolic consequences of HFD and a high‐fructose diet by correcting metabolic and physiological rhythms (Hatori *et al*, 2012; Sun *et al*, 2018). However, the effect of TRF on weight loss varies among diets and experimental protocols and needs to be further verified (Hatori *et al*, 2012; Chaix *et al*, 2014; Woodie *et al*, 2018). Based on findings from *Drosophila* studies, the TRF regimen can enhance the amplitude of oscillating transcripts in the peripheral tissue and head, leading to improved sleep, prevention of body weight gain, and deceleration of cardiac aging (Gill *et al*, 2015). In addition, the efficacy of TRF has been reported in human studies. Food consumption at an inappropriate circadian time has been associated with weight gain and metabolic disorders (Hibi *et al*, 2013). In contrast, an 8‐h TRF at the appropriate time (10:00 to 18:00 h) for 12 weeks reportedly reduced body weight and systolic blood pressure in obese subjects (Gabel *et al*, 2018). Additional studies in humans have shown that TRF in the early phase positively impacts human health, such as improving insulin sensitivity and blood pressure (Sutton *et al*, 2018). These reports suggest that circadian clock remodeling, especially enhancing the clock amplitude, affords positive health effects and maintains appropriate rhythms, critical for preventing obesity and metabolic disease.

## Remodeling of the clock and metabolism by natural compounds

In addition to dietary composition, natural compounds can influence circadian and metabolic homeostasis. Several studies have identified single nutrients capable of driving or phase‐shifting circadian rhythms.

Flavonoids, polyphenolic compounds derived from plants and fungi, reportedly possess numerous biological and pharmacological activities, including anti‐inflammatory, antioxidant, and anticancer effects. Recent studies have revealed that flavonoids have great therapeutic potential for treating diet‐induced obesity and hepatic inflammation, given their ability to manipulate the clock system.

Epigallocatechin gallate (EGCG) is a major component of tea polyphenols. EGCG treatment of hepatocytes exposed to H_2_O_2_ demonstrated that EGCG had a protective effect against H_2_O_2_‐induced circadian misalignment (Qi *et al*, 2018). Another *in vitro* study reported that EGCG supplementation can reverse obesity‐induced insulin resistance and obesity by alleviating circadian desynchrony and metabolic misalignment (Mi *et al*, 2017).

Nobiletin, a polymethoxylated flavone isolated from citrus peels, affords potent protection against metabolic syndromes in a clock‐dependent manner (He *et al*, 2016). Nobiletin targets ROR, which in turn may stabilize and even enhance the transcriptional activity of the whole molecular oscillator. The anti‐obesity effect of nobiletin was diminished in *ClockΔ19*/*Δ19* mutant mice, indicating that nobiletin exerts its metabolic effect through *Clock*. In the muscle, nobiletin reportedly promotes healthy aging by ROR activation under HFD treatment via a concerted optimization of mitochondrial respiration (Nohara *et al*, 2019). Similar effects were observed with tangeretin, a flavonoid present in citrus peels, using the PER2::LUC luciferase system (Shinozaki *et al*, 2017).

Resveratrol (3,4,5‐trihydroxy‐trans‐stilbene) is a natural polyphenolic compound found in several fruits and vegetables, such as grapes and peanuts. Several of the health benefits associated with resveratrol have been attributed to its ability to mimic the effects of calorie restriction. Resveratrol increases intracellular cAMP concentration by inhibiting cAMP phosphodiesterases, enhancing AMPK activity (Park *et al*, 2012). Moreover, resveratrol upregulates not only protein expression but also the enzyme activity of SIRT1 in multicellular animals (Howitz *et al*, 2003; Chai *et al*, 2017). Resveratrol intervention during HFD restored the rhythmic expression of *Sirt1*, concomitant with the restoration of clock genes (Sun *et al*, 2015). Numerous functional components and metabolites of food that impact the clock are likely to be discovered in future investigations.

## Conclusions and future perspectives

During past decades, a large array of nutritional challenge studies have provided insights into the reciprocal relationship between the circadian system and metabolic homeostasis. In addition to dietary composition, macronutrients can influence circadian rhythmicity. However, numerous outstanding questions remain unresolved.

Over the last decade, several circadian transcriptome analyses have been conducted to characterize the circadian and metabolic genes expressed in different organs and under several dietary conditions. Interestingly, the effects of diet differ depending on the organ, which is influenced by tissue‐specific transcription factors. The interaction between organs via output metabolites has also been reported, although a comprehensive understanding is yet to be established. Future research should aim to determine the contribution of each peripheral clock to metabolic homeostasis in other organs.

Recent studies on various diets have revealed the effects of diet composition on clock genes and transcription factors. However, results across studies have been contradictory. As diet compositions vary substantially from study to study, a careful and comprehensive approach is required to elucidate the intricate mechanisms linking the clock and metabolism.

Most studies investigating clock changes and metabolic function changes attributed to dietary modifications have been performed in rodents. Available information regarding the impact of various dietary changes on the human clock system remains insufficient. Blood samples can be obtained using minimally invasive methods and are extremely valuable for extracting important biological information. For example, plasma levels of PC reflect the dietary carbohydrate–fat ratio in humans (Inoue *et al*, 2017). A recently published study has illustrated changes in metabolites in human serum in response to dietary changes over circadian time (Sato *et al*, 2018). Therefore, blood samples can be substantially useful for determining the effect of dietary composition on metabolic rhythms in humans. Further studies on chrono‐nutritional aspects of the circadian clock can provide potential therapeutics for treating metabolic diseases.

## Disclosure and competing interests statement

The authors declare that they have no conflict of interest.

**In need of answers**

- How does each organ’s clock interact with other peripheral clocks?
- How do nutritional challenges impact tissue‐specific remodeling of the clock?
- What is the precise relationship between diet composition‐period and remodeling of the clock?
- How does β‐hydroxy butyrylation impact the clock at chromatin under a ketogenic diet?
- Accurate methods for detecting the nutritional effect on the human circadian system are needed.
